# Postop Complication With Euglycemic Diabetic Ketoacidosis in a Patient Receiving Empagliflozin

**DOI:** 10.7759/cureus.33161

**Published:** 2022-12-31

**Authors:** João Fustiga, Marco Fernandes, Francisca Dâmaso, Joana A Duarte, Catarina Rodrigues

**Affiliations:** 1 Intensive Care Unit, Centro Hospitalar Lisboa Ocidental, Lisbon, PRT; 2 Internal Medicine, Hospital de São Francisco Xavier, Lisbon, PRT; 3 Internal Medicine, Centro Hospitalar Lisboa Ocidental, Lisbon, PRT

**Keywords:** elevated anion gap metabolic acidosis, empagliflozin, sodium-glucose co-transporter 2 inhibitors (sglt2i), diabetic ketoacidosis, diabetes mellitus

## Abstract

Euglycemic diabetic ketoacidosis (EDKA) is an uncommon diabetic complication with increasing prevalence and is associated with the use of sodium-glucose co-transporter 2 inhibitors (SGLT2i).

We report the case of a 77-year-old female patient with type 2 diabetes mellitus, treated with metformin/linagliptin and empagliflozin, who initiated a slurred speech and altered level of consciousness in the postoperative period of a cholecystectomy. On observation, the patient presented with Kussmaul breathing and mucosal dryness and was ketotic. Laboratory exams showed metabolic acidosis with an elevated anion gap, normoglycemia, and positive ketonemia. Fluid replacement with dextrose solution and continuous insulin infusion were initiated, with progressive clinical and laboratory improvement. On discharge, she showed resolution of symptoms, and empagliflozin was discontinued from her usual medication.

Despite the warnings of European and American medical agencies in 2015/2016, EDKA remains a challenging diagnosis due to its unspecific and insidious symptoms.

## Introduction

Sodium-glucose co-transporter 2 inhibitors (SGLT2i) are oral antidiabetic class drugs with rising popularity due to its high safety profile and broad cardio- and nephroprotective effects [1]. SGLT2i have been reported to be associated with an increased risk of urinary and genital tract infections, volume depletion, and bone fractures [2].

Euglycemic diabetic ketoacidosis (EDKA) is a rare complication of diabetes mellitus (DM) characterized by the triad of normoglycemia (serum glucose < 250 mg/dL), elevated anion gap metabolic acidosis (serum bicarbonate < 18 mEq/L, and pH < 7.3), and ketosis (ketones > 0.6 mmol/L) [2,3]. Recent reports have shown a higher predisposition to EDKA in patients treated with SGLT2i, especially with empagliflozin [4]. In this article, we report a case of an elderly woman treated with empagliflozin who presented with EDKA as a postop complication.

## Case presentation

A 77-year-old woman presented in the emergency department with complaints of abdominal pain localized to the right hypochondrium, as well as nausea and vomiting with three days of evolution. She had a known history of DM for over 10 years and was treated with empagliflozin 10 mg once a day and metformin 1000 mg associated with linagliptin 2.5 mg twice a day. Abdominal ultrasound revealed acute lithiasic cholecystitis, and empirical antibiotic treatment with piperacillin-tazobactam was begun.

On the third day of admission and after 12 hours of laparoscopic cholecystectomy, the patient developed slurred speech and prostration. A general physical examination revealed signs of dehydration, Kussmaul breathing, and ketotic breath.

The arterial blood gas analysis displayed severe metabolic acidosis with an elevated anion gap, without hyperlactacidemia. Laboratory studies showed normocytic normochromic anemia, leukocytosis with neutrophilia, and elevated C-reactive protein. Occasional serum glucose was within the normal range. Ionogram had borderline low potassium, with normal sodium and chloride. She also had a prerenal acute kidney injury, with an estimated glomerular filtration rate of 25.81 mL/min/1.73 m^2^. She had poor ambulatory glycemic control, with a hemoglobin A1c of 7.8%. Laboratory results are shown in Tables 1, 2.

**Table 1:** Laboratory findings on first evaluation with normal range results MCV: Mean cell volume; MCH: Mean cell hemoglobin; BUN: Blood urea nitrogen.

Laboratory finding	Initial evaluation	Normal range
Hemoglobin (g/dL)	10.4	12.0–15.0
- MCV (fL)	87.8	80.0–96.1
- MCH (pg)	27.8	27.3–33.7
Leukocytes (x 10^9^/L)	15.0	4.0–10.0
- Neutrophil (%)	88.7	40.0–80.0
- Lymphocyte (%)	6.1	20.0–40.0
- Monocyte (%)	4.9	2.0–11.7
- Eosinophil (%)	0.0	1.0–6.0
- Basophil (%)	0.3	0.0–2.0
Platelets (x 10^9^/L)	303	150–400
Sodium (mmol/L)	141.0	136–145
Potassium (mmol/L)	3.52	3.50–5.10
Chloride (mmol/L)	100.0	98–107
Urea, plasma (BUN) (mmol/L)	9.49	2.9–8.9
Creatinine (mmol/L)	1.85	0.50–0.90
Lipase (U/L)	35	<75
Amylase (U/L)	40	<100
C-reactive protein (g/dL)	41.3	<0.5
Procalcitonin (ng/mL)	3.20	<0.1

**Table 2:** Evolution of arterial blood gas analysis throughout treatment

Arterial blood gas findings	pH	pO_2_ (mmHg)	pCO_2_ (mmHg)	HCO_3_^-^ (mmol/L)	Anion gap (mmol/L)	Lactate (mmol/L)	Glucose (mg/dL)	Ketones (mmol/L)
Initial evaluation	7.19	92.0	40.0	15.0	20.0	0.9	174	7.7
1 hour after starting the treatment	7.206	97.3	38.8	14.8	20.4	0.8	295	5.0
2 hours after starting the treatment	7.242	90.8	44.2	18.4	19.8	0.8	295	2.2
4 hours after starting the treatment	7.233	83.9	52.5	21.3	13.0	1.0	267	1.4
8 hours after starting the treatment	7.347	90.3	44.5	24.3	12.2	0.8	254	1.0
Normal range	7.35–7.45	75.0–100.0	35.0–45.0	21.0–28.0	7.0–16	0.50–2.00	<250	<0.6

As the initial clinical picture showed lethargy associated with an elevated anion gap metabolic acidosis, the diagnosis of diabetic ketoacidosis was suspected. Due to no history of alcohol, NSAIDs, or pain medication in the past, alcoholic or drug-induced ketoacidosis was excluded. Her lactate level was also within normal range excluding lactic acidosis. Pancreatitis was ruled out as serum amylase and lipase were within the standard range. As she presented with normal values of capillary glycemia and high levels of capillary ketonemia (7.7 mg/dL), the diagnosis of EDKA was done. The normal value of C-peptide excluded insulin deficiency.

Insulin infusion and intravenous hydration with dextrose solution, as well as intravenous potassium supplementation, were initiated. The patient presented with progressive improvement of acidosis and bicarbonate (Table 2). Insulin infusion was switched to a basal-bolus regimen, insulin glargine, and insulin lispro. She remained stable, and her symptoms resolved completely. Then, she was discharged home with the discontinuation of empagliflozin.

## Discussion

We present a case of EDKA with multiple triggers identified such as infection (acute lithiasic cholecystitis), surgery (laparoscopic cholecystectomy), prolonged fasting, and empagliflozin intake. Ketotic halitosis, combined with elevated anion gap metabolic acidosis and normoglycemia, was a key clinical finding to suspect diabetic ketoacidosis. As suggested by the literature, a dextrose solution was chosen to prevent hypoglycemia, and insulin was administered to solve ketoacidosis. Potassium supplementation was administered to prevent hypokalemia induced by insulin infusion as it increases cellular uptake of potassium.

Approximately 2.6%-3.2% of diabetic ketoacidosis are euglycemic [5]. The underlying pathophysiology of EDKA includes an absolute or relative insulin deficiency with severe insulin resistance [5-7]. This deficit increases glucagon production and release of free fatty acids, leading to ketogenesis by the production of acetone, β-hydroxybutyrate, and acetoacetic acid [5-7]. β-hydroxybutyrate accounts for 78% of all ketones in the body, which can produce volatile acetone and be present in exhaled breath, creating a characteristic fruity breath called ketotic halitosis [8]. EDKA diagnosis requires the presence of the triad of normoglycemia, elevated anion gap metabolic acidosis, and ketosis [2,3]. Treatment should be immediately initiated with dextrose 5% concurrently with IV insulin, aiming to correct ketosis and prevent carbohydrate starvation [2,3]. Potassium supplementation is recommended with 10 mEq/L IV for those with serum potassium of 3.5-5.5 mEq/L [2,3]. If serum potassium is higher than 5.5 mEq/L, potassium supplementation can be held [2,3]. Serum electrolytes and glucose should be monitored every hour [2,3]. However, delays in diagnosis and treatment can result in severe acidosis, thrombosis, infection, seizures, cerebral edema, hemodynamic compromise, and prolonged hospital length of stay [2,3].

Blau et al. estimated a seven-fold risk increase of diabetic ketosis in type 2 DM patients under SGLT2i [9]. The pathophysiology underlying EDKA is due to an enhanced excretion of glucose by blocking the reabsorption of filtered glucose from the proximal convoluted tubule [7]. The loss of urinary glucose creates a state of carbohydrate starvation and volume depletion, increasing the glucagon/insulin ratio, which in turn results in a life-threatening state of severe dehydration and ketosis [7]. Furthermore, SGLT2i have been found to stimulate the release of glucagon from pancreatic α-cells, suppressing the removal of beta-hydroxybutyrate and acetoacetate by kidneys and thus worsening ketosis [7,8]. A normal C-peptide allows the exclusion of insulin deficiency, thereby reinforcing the diagnosis due to the normal production of insulin by the pancreatic β-cell [8]. Ata et al. identified the infection as the most common trigger for EDKA overall, followed by insulin noncompliance, pancreatitis, and surgery. The main trigger is not identified in most patients [9,10]. It is important to mention that EDKA could also be triggered in DM-2 patients with nonalcoholic fatty liver disease, particularly in older male patients [11]. After the identification of EDKA triggers, SGLT2i should be discontinued [12].

We present the presence of multiple triggers for EDKA as a limitation of this study, which makes it difficult to identify SGLT2i as the main factor. The patient presented with an infection and was submitted to surgery, resulting in acute metabolic stress. Therefore, glucagon release and peripheral insulin resistance increased, further worsening the ketoacidosis status. Recent studies describe women and the presence of SLGT2i polymorphisms as predisposing conditions to a higher occurrence of EDKA, without any strong evidence.

## Conclusions

Patients treated with SGLT2i presenting with prostration and elevated anion gap metabolic acidosis should be tested for the presence of ketones. The early recognition of risk factors in patients under SGLT2i allows the prevention and treatment initiation of EDKA. Standard guidelines should be developed to help physicians in the approach of these patients.
